# Mathematical modelling of interacting mechanisms for hypoxia mediated cell cycle commitment for mesenchymal stromal cells

**DOI:** 10.1186/s12918-018-0560-3

**Published:** 2018-04-02

**Authors:** Bo Zhang, Hua Ye, Aidong Yang

**Affiliations:** 1 Department of Engineering Science, University of Oxford, Oxford, UK; 2 Institute of Biomedical Engineering, Department of Engineering Science, University of Oxford, Oxford, UK

**Keywords:** Intracellular modelling, Hypoxia, Cell cycle commitment, HIF, ROS, Global sensitivity analysis, Mesenchymal stromal cells

## Abstract

**Background:**

Existing experimental data have shown hypoxia to be an important factor affecting the proliferation of mesenchymal stromal cells (MSCs), but the contrasting observations made at various hypoxic levels raise the questions of whether hypoxia accelerates proliferation, and how. On the other hand, in order to meet the increasing demand of MSCs, an optimised bioreactor control strategy is needed to enhance in vitro production.

**Results:**

A comprehensive, single-cell mathematical model has been constructed in this work, which combines cellular oxygen sensing with hypoxia-mediated cell cycle progression to predict cell cycle commitment as a proxy to proliferation rate. With oxygen levels defined for in vitro cell culture, the model predicts enhanced proliferation under intermediate (2–8%) and mild (8–15%) hypoxia and cell quiescence under severe (< 2%) hypoxia. Global sensitivity analysis and quasi-Monte Carlo simulation revealed that within a certain range (+/− 100%), model parameters affect (with varying significance) the minimum commitment time, but the existence of a range of optimal oxygen tension could be preserved with the hypothesized effects of Hif2α and reactive oxygen species (ROS). It appears that Hif2α counteracts Hif1α and ROS-mediated protein deactivation under intermediate hypoxia and normoxia (20%), respectively, to regulate the response of cell cycle commitment to oxygen tension.

**Conclusion:**

Overall, this modelling study offered an integrative framework to capture several interacting mechanisms and allowed in silico analysis of their individual and collective roles in shaping the hypoxia-mediated commitment to cell cycle. The model offers a starting point to the establishment of a suitable mechanism that can satisfactorily explain the different existing experimental observations from different studies, and warrants future extension and dedicated experimental validation to eventually support bioreactor optimisation.

**Electronic supplementary material:**

The online version of this article (10.1186/s12918-018-0560-3) contains supplementary material, which is available to authorized users.

## Background

Hypoxia has been shown to enhance the proliferation of cancer cells, stromal cells and stem cells [1–3], though the exact mechanisms remain to be elucidated. It has been hypothesised that a lower oxygen concentration facilitates cell cycle progression and reduces the generation of reactive oxygen species (ROS) [4–6], which would otherwise incur elevated apoptosis and mutation [7]. On the other hand, experiments have found that hypoxia can drive cells into quiescence to escape from oxidative stress [7–10]. These inconsistent experimental findings in the literature could potentially be attributed to cell pool heterogeneity or different culture conditions. With the increasing market demand for large-scale in vitro stem cell production, it is crucial to establish the definitive impact of hypoxia on stem cell proliferation. In particular, a mathematical model that incorporates the key mechanisms regulating the influence of oxygen concentration on cell cycle can be instrumental, which is currently lacking.

One factor that contributes to the controversy over the effects of hypoxia on cell proliferation is the lack of agreed definition for “hypoxia” and “normoxia” [11]. Physiologically, oxygen concentration in blood is around 5–13%, and is further reduced in tissues [2]. Some reports used oxygen levels of less than 5% as the normoxic oxygen standard [2, 12] to reflect the physiological “in situ normoxia” [13]. Contrastingly, a majority of others used atmospheric oxygen level of 20% to represent a controlled normoxia environment for cell culture in vitro [1, 9, 10]. Additionally, a wide range of oxygen levels have been referred to as hypoxia in cell cultures, from 0.2% - 5% [3, 14–16]. The collective ambiguity in these definitions imposes further challenges in identifying how lower oxygen conditions affect cell proliferation. To avoid confusion, the following ranges defining in vitro cell culture oxygen tensions are used consistently throughout the current paper: severe hypoxia (< 2%), intermediate hypoxia (2% - 8%), mild hypoxia (8% - 15%), normoxia (15% - 20%).

In general, each cell line possesses a unique set of characteristics to govern how it responds to oxidative stress. This work was designated as part of an effort to improve in vitro culture of human mesenchymal stromal cells (MSCs). However, due to the lack of a complete set of protein expression data for MSCs from a single source, results from experimental studies of different cell lines have been used. The mechanisms/systems that are evolutionarily conserved and shared across cell lines are distinguished from those that are distinct in each tissues/species. The basic cell cycle model has been found in yeast, *drosophila*, mammalian cells, etc. [17]. It is believed that the mechanisms are comparable, despite the genetic differences. Hypoxic responses can be split into the downstream actors of hypoxia inducible factor (HIF) isomers, Hif1α and Hif2α. Hif1α expression has been found in most cells and it affects cell metabolism, angiogenesis, cell cycle progression, survival, etc. [18]. Hif1α has also been reported to maintain MSCs pool in a primitive state by allowing selective self-renewal [19]. Hif2α is cell line specific and regulates cell proliferation, vascularization and maintains stem cell characteristics [20]. Intracellular oxygen molecule allows prolyl hydroxylase and 2-oxoglutarate to corroborate in tagging HIF for ubiquitin-dependent degradation [18]. Despite similar mechanisms, the characteristics of this oxygen-dependent degradation are different between the isomers. As a result, the relative protein accumulation under normoxia is different between the two isomers, albeit always much lower than their hypoxic levels.

In the existing literature, most hypoxia-induced quiescence was reported under severe hypoxia [8–10, 21], where the Hif1α level and activities are prominent. Enhanced proliferation was observed under intermediate and severe hypoxia conditions [1–3, 22–25], where the relative strength of Hif2α overshadows that of Hif1α. The differential responses of Hif1α and Hif2α to oxygen level are believed to be key in explaining seemingly contradictory observations with respect to cell cycles under the broadly defined hypoxia conditions [6, 11]. Other hypoxia-mediated factors (i.e., Notch [26]) are not captured in this work because of a missing consensus on their impact on cell cycle progression.

Various sophisticated models [27–29] have been proposed to predict the level of Hif1α protein in response to cellular oxygen tension, which are continuously evolving as more mechanisms are discovered. On the other hand, an exponential decay model has been used as a proxy for a simplified estimation of total Hif1α protein as a function of oxygen tension [27, 30], which is also adopted in this work.

A proliferating cell commits to cell cycle when it passes the restriction point in late G1 phase, which is a process that has been represented by a few existing models. Bedessem and Stephanou [30] established a model describing how hypoxia prolongs cell cycle commitment, expanding on Novak and Tyson’s work [17]. The effect of oxygen on cell cycle was linked through Hif1α protein. Hif1α was assumed to have a direct inhibitory effect on cyclin D, based on the findings from Wen et al. [16]. Threshold levels for cyclin E and SCF, a cyclin E antagonist, were set to collectively mark G1-S phase transition. The model showed that the time required by the commitment to cell cycle increases as oxygen tension decreases. At below 0.06% oxygen, cell quiescence was reached. The Hif1α estimation is only applicable in the case of up to 6% oxygen. The authors acknowledged the activation effect of Hif2α on cyclin D but only focused on the effect of Hif1α in their model.

Dong et al. [31] modelled the effect of E2F level on cell cycle entry with experimental validation. More than 100 single-cell analyses were performed to correlate cell proliferation status against their E2F level. The level of E2F was found to govern G1-S phase commitment, whereas the levels of cyclins D and E and Myc affect the time required to reach the transition point. Different Myc-inhibitors were tested to indicate the significance of Myc protein in regulating cell cycle. The model presented in Dong et al. [31] incorporates the cumulative findings from the previous simulation and experimental discoveries by their group [32, 33]. The effect of CDKI (cyclin dependent kinase inhibitor) and possible linkage to hypoxic responses were not addressed in the study.

Another approach in modelling proliferation has been associated with cellular metabolism and the consideration of nutrient supplies [34]. HIF alters cellular metabolic pathways and nutrient uptakes under hypoxia [35]. The current study does not include the metabolic impacts on proliferation and assumes the nutrient supply to be always in excess. Other HIF-mediated hypoxic regulation of proliferation has been reported [36] but the incorporation of these pathways is beyond the scope of this study.

With the purpose of enhancing stem cell proliferation, this work aims to distinguish the effects of hypoxia on cell proliferation qualitatively and quantitatively, through modelling the protein-level variations. To this end, models described in the literature mentioned above, although very relevant, need to be further enhanced particularly in two respects, namely (i) incorporating both Hif1α and Hif2α into the cell cycle model to encompass their distinctive roles, and (ii) connecting the hypoxia sensing model with the cell cycle model, through Myc protein, to enable the direct prediction of the effect of hypoxia level on cell cycle commitment. The intention of this work is to build such an improved model and use it to (i) make predictions of the impact of hypoxia on cell proliferation over the whole range of relevant oxygen concentration levels, against various experimental observations as introduced earlier, (ii) elucidate the likely roles of parallel mechanisms regulating cell cycle progression that underpin the predicted and observed behaviours, and (iii) identify significant model parameters and hypotheses that deserve special attention of further investigation. The learning from this work thus may hold the potential to form a foundation of future research for enhancing the in vitro culturing efficiency of MSCs and other cell lines by the optimal control of oxygen concentration in bioreactors.

## Methods

**Fig. 1:**
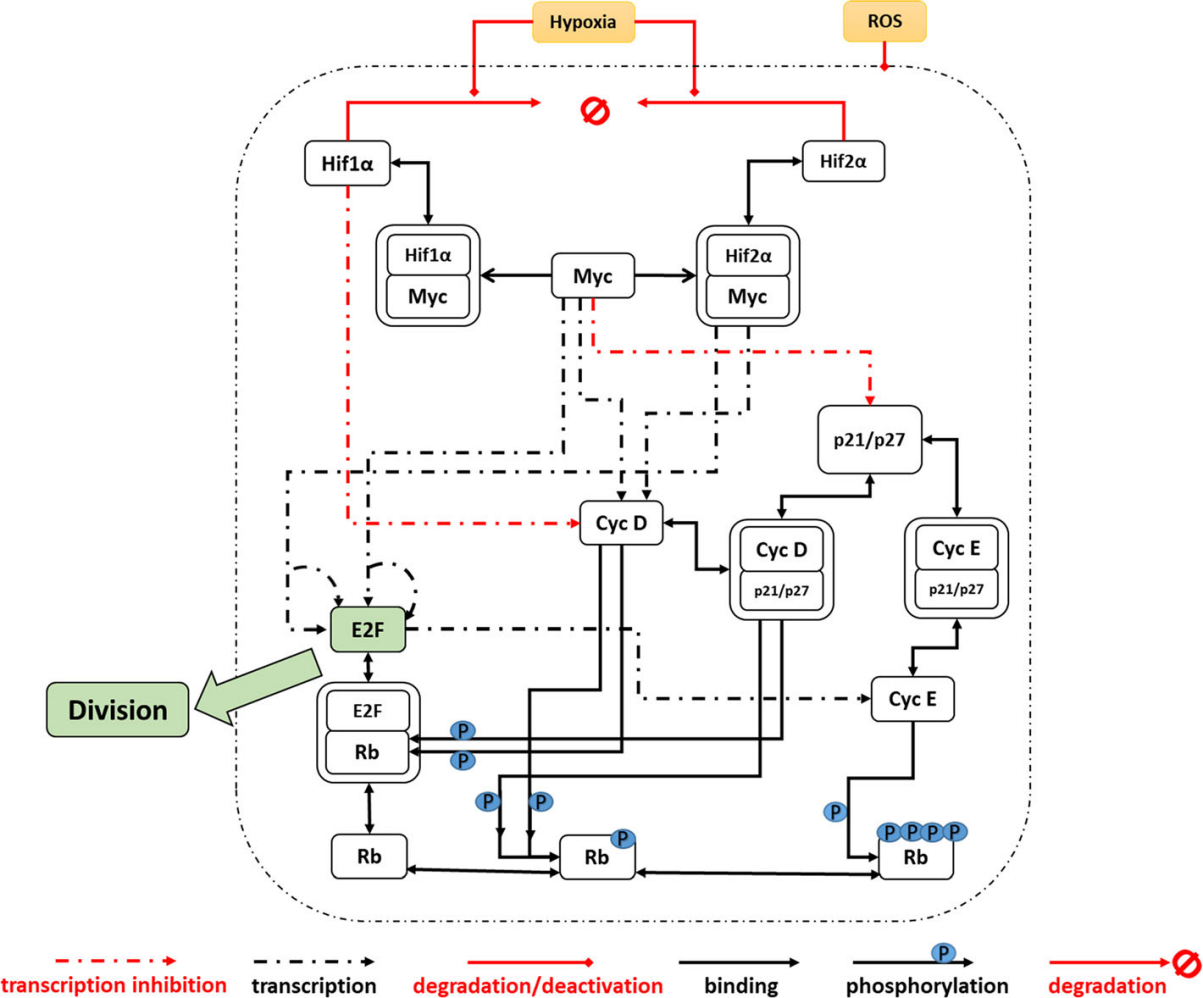
Assumed simplified mechanisms for the effects of hypoxia on cell cycle commitment. Hypoxia varies the active amount of Hif1α and Hif2α proteins, through which cell cycle regulating proteins are regulated. The variation in cell cycle regulators positively (cyclin D, cyclin E, Myc) or negatively (p21/p27 and Rb) controls the downstream protein level of E2F. The progression of cell cycle G1-phase into S-phase commitment is controlled by the accumulation of E2F protein exceeding a set threshold level. Different biological interactions are shown in the figure

A model to quantitatively reflect how hypoxia affects cell proliferation has been constructed (Fig. 1). The key features of the model include linking hypoxia sensing and cell cycle progression. Furthermore, Myc protein is used as the primary intermediate actor to correlate HIF protein levels to various cell cycle regulators. Hif1α and Hif2α physically bind to Myc to inhibit or enhance its activity, respectively. As the availability of two HIF isoforms varies with oxygen concentration, the effective Myc level is affected and results in the change in the cell proliferation status. A direct transcriptional inhibition of Hif1α on cyclin D is included [16, 30].

The current model aims to capture the effects of oxygen content in regulating the time required for a proliferating cell to commit to progressing past the restriction point, after which the process of cell division becomes irreversible. The model considers a hypoxic culture condition with a specific oxygen level which determines the concentrations of HIF. To focus on key mechanisms and maintain simplicity, assumptions were made in several parts of the model, in comparison with the more detailed treatments available in the literature [28]. Non-stem-cell data was used to calibrate Hif1α and cell cycle models, as they appear to be common among different cell types. No literature evidence was found to question the validity of using these data for stem cell modelling. Stem cells data on Hif2α expression were obtained from the literature.

The model equations are presented in the following sections. In general, the mechanisms for protein-protein binding, transcription and phosphorylation are represented by the Hill function to capture the sigmoidal or hyperbolic behaviour. The behaviours that are regulated by more than one factor (e.g., the Myc-dependent auto-catalytic transcription of E2F) are captured by multiplying the relevant terms. Protein-protein interactions are described with mass-action kinetics, consistent with literature models [31, 33]. In these equations, variables in brackets represent concentrations of various species (including proteins and oxygen). “k”, “m” and “n” are used to denote different types of constants, which are further distinguished by subscripts. “kf” and “kr” are the respective forward and reverse rate constants. “g” indicates the basal generation rate and “d” represents the basal degradation rate of respective proteins. Protein-to-protein binding is represented by “-” that connects two proteins. “DEG” is a parameter that represents a basal protein deactivation and degradation mechanism. Exponential and absolute value functions are expressed by “exp” and “abs”, respectively.

Overall, the hypoxia sensing – cell cycle progression model consists of 13 ODEs and 3 algebraic correlation equations (Eqs. 1, 2 and 8). The parameters and initial conditions were obtained or adjusted from literature data, as shown in the Supporting Information (Additional file 1). Unless stated otherwise, these values have been taken as nominal values in the simulation studies reported in this work.

### HIF modelling

An exponential expression is used in place of the more complex depiction to estimate the available HIF protein. Bedessem and Stephanou [30] and Dayan et al. [27] applied such an approach to estimate the total amount of Hif1α protein at different oxygen concentrations. Experimental data from six different cell lines from Bracken et al. [37] confirms the validity of the exponential relationship. It is further assumed that all available Hif1α proteins are functionally active and nucleus-bound. Equations 1 and 2 estimate the level of Hif1α and Hif2α proteins, respectively. “t_i_” is the normalization oxygen level and “p_i_” is the oxygen level where HIF protein peaks, *i* = 1 or 2.

1 $$ \left[\boldsymbol{Hif}\mathbf{1}\boldsymbol{\alpha} \right]=m1\ast \mathit{\exp}\ \left(b1\ast \left(1- abs\ \left(p1\hbox{--} \left[{O}_2\right]\right)/t1\right)\right) $$

2 $$ \left[\boldsymbol{Hif}\mathbf{2}\boldsymbol{\alpha} \right]=m2\ast \mathit{\exp}\ \left(b2\ast \left(1- abs\ \left(p2\hbox{--} \left[{O}_2\right]\right)/t2\right)\right) $$

**Fig. 2:**
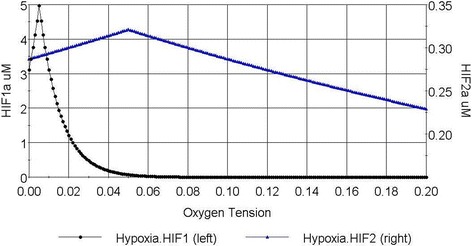
Simulated expression of Hif1α and Hif2α at different oxygen levels from this study. Hif1α (black) level corresponds to the primary axis. Hif2α (blue) level corresponds to the secondary axis

Jiang et al. [15] reported the measured Hif1α level for 0.5–10% oxygen, which was used for model calibration. A decline in Hif1α level at below 0.5% was also observed [15]. Various reports found that Hif1α protein was undetectable at greater than 5% oxygen in stem cells [3, 14]; Bracken et al. reported that Hif1α protein became negligible at higher than 10% oxygen in various cell lines [37]. In our model, the operating range for Hif1α is set at 0.5%–10% oxygen with an exponential decline behaviour. The simulation result is shown in Fig. 2. The maximum Hif1α concentration is assumed to be 5 μM, using data reported from abcam [38]. Note that Hif1α protein has been detected under atmospheric oxygen condition; this phenomenon may be cell line or medium specific [37], and will be accounted for when a complete, comprehensive data set becomes available in the future.

No existing model for the response of Hif2α to oxygen level was found in the literature. Hif2α is thus assumed to behave in the same exponential fashion as Hif1α. In stem cells, Hif2α protein was found to peak at oxygen levels between 2 and 5%, higher than that of Hif1α [6]. The model sets a peak value for Hif2α protein at 5% oxygen. At below 5% oxygen, Hif2α protein level is assumed to mirror its behaviour at greater than 5%. Hif2α proteins are detectable in cells cultured under 20% oxygen [3, 14]; therefore, the exponential decline is assumed to be valid for the entire range of the simulated oxygen level. The expression level of Hif2α appears to vary significantly across cell lines, therefore it is necessary to use stem cell data for calibrating its model. The western blot data of human Embryonic stem cells (hESCs) from Forristal et al. [3] distinguished the relative expression of Hif2α protein between 5% and 20% oxygen, while Hif1α level was not detectable at 5%. Narva et al. [14] measured the relative level of Hif1α and Hif2α proteins at 4% oxygen. The relative ratios acquired from the two papers were used to derive the exponential decay model of Hif2α with respect to oxygen. The simulated trend of Hif2α protein level is shown in Fig. 2.

### Cell cycle modelling

A cell cycle model was built on the basis of several published models in the literature [17, 30, 31, 39]. The model describes the interactions between Myc, CDK, cell cycle regulatory protein-Rb, cyclins and cell cycle commitment determinant protein-E2F, as shown in Fig. 1. Cell cycle is modelled up to the restriction point (RP) in late-G1 phase, beyond which cell proliferation is imminent. Different basal generation and degradation rates are applied to different proteins.

The binding of Hif1α and Hif2α to Myc respectively alters its activity negatively or positively. The activity of Myc and Hif1α alters downstream accumulation of cell cycle regulators. Previous models used different identifiers to indicate progression past RP (or commitment to S-phase). The exceeding of a target E2F concentration is used in our model to indicate commitment to cell cycle, as confirmed with simulation and experimental results in Dong et al. [31]. Yao et al. [33] reported the limit of 1 μM E2F concentration in turning the cell “on” to proliferate. The duration required for cells to reach the E2F threshold, termed “commitment time” thereafter, is dependent on the oxygen level via the levels of HIFs, governed by the following equations (Eqs. 3–7).

3 $$ \frac{\boldsymbol{d}\left[\boldsymbol{Hif}\mathbf{1}\boldsymbol{a}-\boldsymbol{Myc}\right]}{\boldsymbol{d}\boldsymbol{t}}=\hbox{--} kr3A\bullet \left[ Hif1a- Myc\right]+ kf3A\bullet \left[ Hif1a\right]\bullet \left[ Myc\right]\hbox{--} {d}_{Hif1a\hbox{--} Myc}\bullet \left[ Hif1a- Myc\right]\bullet DEG $$

4 $$ \frac{\boldsymbol{d}\left[\boldsymbol{Hif}\mathbf{2}\boldsymbol{a}-\boldsymbol{Myc}\right]}{\boldsymbol{d}\boldsymbol{t}}=\hbox{--} kr3B\bullet \left[ Hif2a- Myc\right]+ kf3B\bullet \left[ Hif2a\right]\bullet \left[ Myc\right]\hbox{--} {d}_{Hif2a\hbox{--} Myc}\bullet \left[ Hif2a- Myc\right]\bullet DEG $$

5 $$ \frac{\boldsymbol{d}\left[\boldsymbol{Myc}\right]}{\boldsymbol{d}\boldsymbol{t}}\kern0.5em ={g}_{Myc}+ kr3A\bullet \left[ Hif1a- Myc\right]\hbox{--} kf3A\bullet \left[ Hif1a\right]\bullet \left[ Myc\right]+ kr3B\bullet \left[ Hif2a- Myc\right]\hbox{--} kf3B\bullet \left[ Hif2a\right]\bullet \left[ Myc\right]\hbox{--} {d}_{Myc}\bullet \left[ Myc\right]\bullet DEG $$

6 $$ \frac{\boldsymbol{d}\left[\boldsymbol{cycE}\right]}{\boldsymbol{d}\boldsymbol{t}}= kr12\bullet \left[ cycE-p/p\right]\hbox{--} kf12\bullet \left[ cycE\right]\bullet \left[p/p\right]+{m}_9\bullet \frac{\left[E2F\right]}{\left[E2F\right]+k9}-{d}_{cycE}\bullet \left[ cycE\right]\bullet DEG $$

7 $$ \frac{\boldsymbol{d}\left[\boldsymbol{E}\mathbf{2}\boldsymbol{F}\right]}{\boldsymbol{d}\boldsymbol{t}}={m}_{13}\bullet \frac{\left[ Myc\right]}{\left[ Myc\right]+k13}\bullet \frac{\left[E2F\right]}{\left[E2F\right]+{k}^{\prime }13}+{m}_{E2F}\bullet \frac{\left[ Myc\right]}{\left[ Myc\right]+{k}_{E2F}}+{m}_{13}\bullet \varepsilon \bullet \frac{\left[ Hif2a- Myc\right]}{\left[ Hif2a- Myc\right]+k13}\bullet \frac{\left[E2F\right]}{\left[E2F\right]+{k}^{\prime }13}+{m}_{E2F}\bullet \frac{\left[ Hif2a- Myc\right]}{\left[ Hif2a- Myc\right]+{k}_{E2F}}+{m}_{E2F- RbP}\bullet \frac{\left[E2F- RB\right]\bullet \left(\left[ cycD\right]+\left[ cycD-p/p\right]\right)}{\left[E2F- RB\right]+{k}_{E2F- RbP}}\hbox{--} {k}_8\bullet \left[ RB\right]\bullet \left[E2F\right]-{d}_{E2F}\bullet \left[E2F\right]\bullet DEG $$

Among the CDKIs, p21 and p27 are used in the model to represent the CIP/KIP family of its inhibitory action on cyclin D-CDK4/6 and cyclin E-CDK2. In the literature, different side functions for p21 and p27 were reported [40]. Our model only focuses on the shared cyclin-CDK inactivation of the two proteins. Owing to the much shared commonality between cancer cells and stem cells, a cell mass-independent p27 production rate, previously applied to cancer cells [39], is assumed. INK4 family is another type of CDKI with specific inhibitory effect on cyclin D-dependent kinase [41], which however can be captured through the function of p21/p27 and thus is not included as a separate mechanism in this model.

The concentrations of CDK2, CDK4 and CDK6 do not vary throughout cell cycle and are not expected to be rate limiting [31]. The cyclins are introduced in the model to represent the respective cyclin-CDK dimers. The three different isoforms of cyclin D (cyclin D1, D2 and D3) are not distinguished in the model for simplicity. Cyclin D and cyclin E carry out their functions through Rb phosphorylation [17, 31] and p21/p27 inactivation. Cyclin D physically binds to p21/p27 and deters it from inactivating cyclin E [42]. Equation 8 captures the effect of Hif1α, Myc and Hif2α-Myc on cyclin D transcription, alongside the binding effect of p21/p27. Different factors regulating p21/p27 (denoted by “p/p”) and the associated complexes are shown by Eqs. 9, 10 and 11. The complex of cyclin D-p21/p27 is assumed to share the same activity as cyclin D for Rb phosphorylation [43].

8 $$ \frac{\boldsymbol{d}\left[\boldsymbol{cycD}\right]}{\boldsymbol{d}\boldsymbol{t}}={g}_{cycD}\bullet \left(1-\frac{\left[ Hif1a\right]}{\left[ Hif1a\right]+k3a}\right)\hbox{--} kf6B\bullet \left[p/p\right]\bullet \left[ cycD\right]+ kr6B\bullet \left[ cycD-p/p\right]+{m}_{5 cycD}\bullet \varepsilon \bullet \frac{\left[ Hif2a- Myc\right]}{\left[ Hif2a- Myc\right]+{k}_{5 cycD}}+{m}_{5 cycD}\bullet \frac{\left[ Myc\right]}{\left[ Myc\right]+{k}_{5 cycD}}\hbox{--} {d}_{cycD}\bullet \left[ cycD\right]\bullet DEG $$

9 $$ \frac{\boldsymbol{d}\left[\boldsymbol{p}/\boldsymbol{p}\right]\ }{\boldsymbol{d}\boldsymbol{t}}={g}_{p/p}\bullet \left(1\hbox{--} k4\bullet \left[ Myc\right]\right)- kf6B\bullet \left[p/p\right]\bullet \left[ cycD\right]+ kr6B\bullet \left[ cycD-p/p\right]\hbox{--} kf12\bullet \left[p/p\right]\bullet \left[ cycE\right]+ kr12\bullet \left[ cycE-p/p\right]\hbox{--} {d}_{p/p}\bullet \left[p/p\right]\bullet DEG $$

10 $$ \frac{\boldsymbol{d}\left[\boldsymbol{cycD}-\boldsymbol{p}/\boldsymbol{p}\right]}{\boldsymbol{d}\boldsymbol{t}}= kf6B\bullet \left[p/p\right]\bullet \left[ cycD\right]\hbox{--} kr6B\bullet \left[ cycD-p/p\right]\hbox{--} {d}_{cycD-p/p}\bullet \left[ cycD-p/p\right]\bullet DEG $$

11 $$ \frac{\boldsymbol{d}\left[\boldsymbol{cycE}-\boldsymbol{p}/\boldsymbol{p}\right]}{\boldsymbol{d}\boldsymbol{t}}= kf12\bullet \left[p/p\right]\bullet \left[ cycE\right]\hbox{--} kr12\bullet \left[ cycE-p/p\right]\hbox{--} {d}_{cycE-p/p}\bullet \left[ cycE-p/p\right]\bullet DEG $$

The mechanisms associated with Rb activities are based on Dong et al. [31]. Free Rb protein binds to E2F and inhibits its downstream transcription. Rb is inactivated through cyclin D or cyclin E-dependent phosphorylation. Cyclin D can also dissociate E2F-Rb complex through Rb phosphorylation [31, 33]. Equations 12 and 13 quantify the responses of Rb and E2F-Rb complex, and incorporate the mechanisms of cyclin D-dependent phosphorylation and Rb-to-E2F binding.

12 $$ \frac{\boldsymbol{d}\left[\boldsymbol{Rb}\right]}{\boldsymbol{d}\boldsymbol{t}}={g}_{Rb}\hbox{--} {m}_{cycD- Rb}\bullet \frac{\left(\left[ cycD\right]+\left[ cycD-p/p\right]\ \right)\bullet \left[ Rb\right]}{\left[ RB\right]+{k}_{cycD- Rb}}+{m}_{Rb P}\bullet \frac{\left[ Rb P\right]}{\left[ Rb P\right]+{k}_{Rb P}}\hbox{--} k8\bullet \left[ Rb\right]\bullet \left[E2F\right]-{d}_{Rb}\bullet \left[ Rb\right]\bullet DEG $$

13 $$ \frac{\boldsymbol{d}\left[\boldsymbol{E}\mathbf{2}\boldsymbol{F}-\boldsymbol{Rb}\right]}{\boldsymbol{d}\boldsymbol{t}}=k8\bullet \left[ Rb\right]\bullet \left[E2F\right]-{m}_{E2F- Rb P}\bullet \frac{\left[E2F- Rb\right]\bullet \Big(\left[ cycD\right]+\left[ cycD-p/p\Big)\right]}{\left[E2F- Rb\right]+{k}_{E2F- Rb P}}-{d}_{E2F- Rb P}\bullet \left[E2F- Rb P\right]\bullet DEG $$

Hypo-phosphorylation of Rb is assumed to be carried out only by cyclin D; subsequent cyclin E-dependent hyper-phosphorylation can then take place. Different levels of hyper-phosphorylation have been claimed in the literature; our model assumes a maximum binding of 4 phosphate groups onto Rb. Equations 14 and 15 quantify the change in the concentration of hypo-phosphorylated and hyper-phosphorylate Rb, respectively.

14 $$ \frac{\boldsymbol{d}\left[\boldsymbol{RbP}\right]}{\boldsymbol{d}\boldsymbol{t}}={m}_{cycD- Rb}\bullet \frac{\left(\left[ cycD\right]+\left[ cycD-p.p\right]\ \right)\bullet \left[ Rb\right]}{\left[ Rb\right]+{k}_{cycD- Rb}}\hbox{--} {m}_{cycE- Rb P}\bullet \frac{\left[ cycE\right]\bullet \left(\left[ Rb P\right]\right)\hat{\mkern6mu} \left(n-1\right)}{\left(\left[ Rb P\right]\right)\hat{\mkern6mu} \left(n-1\right)+{k}_{cycE- Rb P}}\hbox{--} {m}_{Rb P}\bullet \frac{\left[ Rb P\right]}{\left[ Rb P\right]+{k}_{Rb P}}+{m}_{E2F- Rb P}\bullet \frac{\left[E2F- RB\right]\bullet \left(\left[ cycD\right]+\left[ cycD-p/p\right]\right)}{\left[E2F- RB\right]+{k}_{cycE- Rb P}}+{m}_{Rb- nP}\bullet \frac{\left[ Rb- nP\right]\hat{\mkern6mu} \left(n-1\right)}{{\left[ Rb- nP\right]}^{n-1}+{k}_{Rb- nP}}-{d}_{Rb P}\bullet \left[ Rb P\right]\bullet DEG\kern1.75em \left(n=4\right) $$

15 $$ \frac{\boldsymbol{d}\left[\boldsymbol{Rb}-\boldsymbol{nP}\right]}{\boldsymbol{d}\boldsymbol{t}}={m}_{cycE- RbP}\bullet \frac{\left[ cycE\right]\bullet \left(\left[ Rb P\right]\right)\hat{\mkern6mu} \left(n-1\right)}{\left(\left[ Rb P\right]\right)\hat{\mkern6mu} \left(n-1\right)+{k}_{cycE- RbP}}-{m}_{Rb- nP}\bullet \frac{\left[ Rb- nP\right]\hat{\mkern6mu} \left(n-1\right)}{\left[ Rb- nP\right]\hat{\mkern6mu} \left(n-1\right)+{k}_{Rb- nP}}\kern2em \left(n=4\right) $$

Different isoforms of E2F are not considered in the model, for simplicity. Our model allows the total concentration of different E2F-involving species to be dynamically varied (Eq. 14) [31, 33], unlike the conservation of individual E2F-involving species assumed by Novak and Tyson and Bedessem and Stephanou [17, 30].

Previous models used different identifiers to indicate progression past RP (or commitment to S-phase). The exceeding of a target E2F concentration is used in our model to indicate commitment to cell cycle, as confirmed by simulation and experimental results in Dong et al. [31]. Yao et al. [33] reported the limit of 1 μM E2F concentration in turning the cell “on” to proliferate. The results from Dong et al. [31] and Yao et al. [33] showed that a 0.66 μM E2F would lead 75% of cells committing to proliferation. The duration required for cells to reach E2F threshold is simulated at different oxygen conditions.

Other models have correlated the basal generation rates of certain cell cycle species to cell mass [30, 39]. The mass-dependent synthesis is not yet incorporated in our model, as it currently focuses on commitment time only and does not offer a full account of cell growth.

### Modelling of protein deactivation

A first-order basal degradation rate is inferred from the literature for each expressed protein. This work assumes an additional ROS-mediated protein deactivation as a function of oxygen tension beyond a threshold oxygen level, which includes proteolytic protein degradation, alteration of protein orientation, oxidation of functional groups and other interactions that remove proteins from actively engaging in reactions. ROS is a collection of reactive species (i.e. superoxide, hydroxyl radicals, etc.) generated as by-products of respiration in mitochondria, as a function of local oxygen level [44, 45]. Enzymes, such as superoxide dismutase (SOD), combat superoxides to reduce cell senescence and ageing [46]. Superoxides are reduced by these enzymes to H_2_O_2_ or, in the presence of reduced transitional metal, to hydroxyl radicals [47]. Compared to niche-grown stem cells, normoxically cultured cells experience elevated pericellular oxygen tension which results in greater ROS generation and reduced ROS metabolism [48]. An elevated intracellular ROS level facilitates protein degradation through an ATP-independent and calcium-independent proteolytic pathway [49].

At an oxygen level below a certain threshold, ROS level is thought to be negligible due to the limited intracellular oxygen availability. However, various literature reports have demonstrated higher ROS levels between 1 and 3% oxygen [50, 51]. This may be credited to additional ROS production mechanisms under severe hypoxia [45, 52]. Under intermediate and severe hypoxia, superoxides are recognized to facilitate Hif1α stabilization and the induction of hypoxic responses [51, 53]. Due to insufficient understanding and high ROS detection variability, this ROS-mediated Hif1α stabilization has not been included in the model. Instead, Hif1α activity is thought to be solely dependent on oxygen tension.

An accurate quantitative correlation between oxygen tension and the overall intracellular ROS level has not been established in literature. This work assumes that a threshold oxygen level (*[O*_*2*_*]*_*TH*_ = 10%) may exist, beyond which superoxide production surpasses the neutralising capacity of combating enzymes and results in efflux out of mitochondria and influx to other cellular compartments [44]. If oxygen tension exceeds this level, the amount of intracellular ROS surpasses the cell’s inherent anti-oxidant capacity and initiates ROS-mediated deactivation mechanism across all proteins. Different activation levels have been tested in this study. An exponential decay function is assumed for this general deactivation mechanism (Eq. 16), where *n_deg* is an adjustable parameter.

16 $$ DEG=\mathit{\exp}\left(n\_\mathit{\deg}\ast \left(\left[{O}_2\right]\hbox{--} {\left[{O}_2\right]}_{TH}\right)/{\left[{O}_2\right]}_{TH}\ \right)\ \left( if\ \left[O2\right]>10\%\right) $$

### Analysis with different parametrisation

Sensitivity analysis was performed to capture the significance of model parameters within the range of +/− 100%. In particular, the derivative-based global sensitivity measures (DGSM) approach [54] was adopted to identify the most prominent parameters in regulating model output independently and interactively. The principle of DGSM can be described by Eqs. 17–19 [55]:

17 $$ {E}_i=\frac{\partial f}{\partial {x}_i}=\frac{f\left({x}_i\ast \left(1+d\%\right)\right)-f\left({x}_i\right)}{d\%} $$

18 $$ {g}_i={\int}_{H^n}{E}_i^2 dx $$

19 $$ {G}_i={g}_i/\left(\ {\sum}_{i=1}^n{g}_i\ \right) $$

In the above equations, E_i_ calculates the local derivatives of the model output *f* (which is the commitment time, CT, in this case) with respect to the i^th^ parameter (*x*_*i*_) in a n-dimensional parameter space (denoted as H^n^), where n is the number of studied parameters. g_i_ represents an averaged value of E_i_^2^ over the space H^n^. Finally, the value of G_i_ (referred to as G-score) indicates the relative significance of parameter i on impacting the simulation outcome. The integration in Eq. 18 can be approximated by using a quasi-Monte Carlo (QMC) sampling method [47]; this work has adopted particularly the Sobol sequence [55] with an incremental sampling size of 500 in the course of obtaining a converged value for a G-score. In order to reduce the computational cost, a preliminary screening based on local sensitivities was used to pick out a subset of parameters, which demonstrated greater impact on the simulation output and were therefore subjected to further analysis by DGSM.

In addition to support DGSM, the set of (parameter value) samples generated by the QMC method has also been adopted for carrying out simulations to assess to what extent the predicted minimum CT and optimal oxygen level vary with the parameter values adopted. This set of simulations is referred to as QMC simulation (named after how the parameter values are sampled). The simulation results have also been used to study the variation in the shape of the curve of CT vs. oxygen level, referred to as the “characterisation curve” in this work, which is quantified through a factor k according to Eq. 20:

20 $$ k=\frac{CT_{norm}-{CT}_{min}}{20\%-{O_2}_{eq}} $$

where CT_norm_ and CT_min_ are the CT corresponding to normoxia (i.e 20% O2) and the minimum CT, respectively; O_2eq_ is the hypoxic oxygen level at which the CT is identical to CT_norm_.

## Results

Dynamic numerical simulations revealed the temporal change in concentrations of key cell cycle regulators for a specified oxygen level. The CT was found to vary with a change in oxygen level. A “U-shape” characterisation curve has been discovered from simulation results, which offers a hypothesis that could potentially explain the varied experimental observations reported in literature. The sensitivity analysis highlighted the relative significance of the model parameters under different oxygen tensions. For each set of parameter values, the oxygen level that results in the lowest CT is termed the optimal oxygen level (*O*_*2-optimal*_). The persistency of the existence of an optimal oxygen level (*O*_*2-optimal*_) is demonstrated by the QMC simulation results. The shape of the characterisation curve was generally preserved but appeared sensitive to the degree of ROS-mediated protein deactivation. Hif2α was found to play a crucial role in facilitating commitment to cell cycle under different gradients of hypoxia.

### Dynamic behaviours

**Fig. 3:**
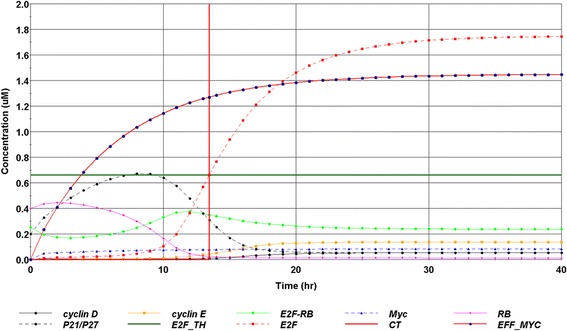
Simulation results showing the dynamic change of selected cell cycle regulators under 2% oxygen. This simulation was completed with the nominal model parameter setting. E2F threshold level (E2F_TH) is shown as the horizontal forest green line. The vertical red line indicates commitment time marking the time when E2F level exceeds the threshold level

Figure 3 shows an example of the simulation output for key cell cycle regulators, at 2% oxygen. It should be noted that, although the model presented above could mathematically predict steady states, they do not carry any biological significance. The useful output of the simulation is only the transient behaviour leading to the accumulation of E2F towards its threshold level, which is reached at 13.5 h in the case shown in Fig. 3. The effective Myc rises steadily, contributed by the basal generation of Myc and the formation of Hif2α-Myc. p21/p27 level rises initially until the Myc-dependent inhibitory effect becomes dominant. Cyclin D is kept at a sustained low level due to the direct inhibition from Hif1α. Rb increases initially due to the disintegration of the pre-existing E2F-Rb dimers. As more E2F is generated, the synthesis rate of Rb-E2F complex increases until all the available Rb is consumed. E2F experiences the initial lag phase due to Rb binding. The sharp sigmoidal behaviour of E2F can be attributed to its Myc-dependent, auto-catalytic mechanism. The level of Cyclin E rises trailing that of E2F, as a result of the E2F-dependent transcription. The trajectories of E2F and cyclin E share the same sigmoidal behaviour. Cyclin E was used as the marker for proliferation identification in Bedessem and Stephanou [30], which would yield a comparable CT according to Fig. 3, also consistent with the range shown in Dong et al. [31].

The behaviour shown in Fig. 3 was predicted using the (default) initial conditions (presented in the Supporting Information (Additional file 1)). To reveal the potential impact of the initial conditions on the qualitative behaviour of the system, dynamic simulations with different initial conditions sampled from a hypercube space formed by +/− 100% of the default initial condition values were carried out, for each of the parameter value sets similarly sampled from a parameters space (details are given in the Supporting Information (Additional file 2)), and across the entire range of oxygen levels. In this analysis, the steady states predicted by the simulations were recorded which, although not carrying any biological significance in themselves as stated earlier, were used to indicate alternative trajectories of the transient behaviour leading to the threshold level of E2F that could arise from different initial conditions. It was found that multiple steady states are indeed present for the combination of a sub range of oxygen levels and a subset of parameter values, but these cases (where a steady state different from that from the default initial conditions was reached) represent only a small potion (less than 2%) of all the simulations carried out. Therefore, model predictions with the default initial conditions are considered to be able to reveal the representative behaviour of the system, and hence have been used for the analysis reported in this work.

### The effect of oxygen level on CT

**Fig. 4:**
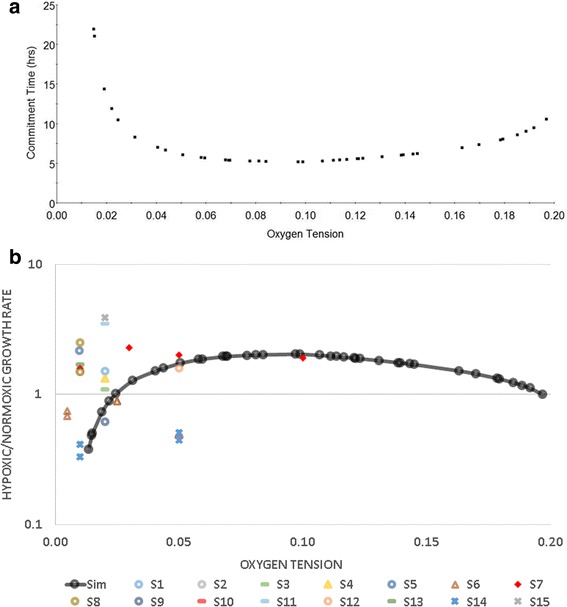
**a** Simulated cell cycle commitment time under different oxygen tensions at the nominal model parameter setting. **b** Compiled literature experimental data of the hypoxic proliferation rate. The ratio between hypoxic and normoxic proliferation rate for each set of the literature data (S1-S15) is plotted. A list of the corresponding references is included in the Supporting Information (Additional file 3). The simulation results from this study (Sim) are shown for the swept oxygen tensions

Simulated CT results under different oxygen concentrations are shown in Fig. 4a. A lower hypoxic CT indicates an enhanced proliferative behaviour, whereas, a greater CT corresponds to a prolonged period till cell cycle commitment. With a culture oxygen level under 2.9%, E2F accumulates much slower, as the inhibitory effect of Hif1α on Myc and cyclin D begins to dominate cell cycle regulation. More severe hypoxia further exacerbates E2F accumulation and leads to cell quiescence at less than 1.2% oxygen.

When the oxygen level is greater than 2.9%, the inhibitory activity of Hif1α is hampered. Myc proteins are less inhibited by Hif1α binding and more E2F and cyclin D are produced. The Hif2α-Myc complex and Myc contribute to E2F availability directly through transcription, and indirectly, through enhancing the production of cyclin D protein for Rb inactivation. Shortened protein accumulation and cell division time under mild hypoxia are revealed from the simulation. The difference in CT is small in the mid-range of oxygen concentration. As oxygen tension rises into the normoxia regime, the effect of ROS-mediated protein deactivation becomes more pronounced reducing the accumulation rate of proliferation determining proteins, which is reflected as ascending CT values. Overall, a minimum CT lies between 8 and 10% oxygen, which is the optimum oxygen concentration for commitment to cell cycle, with the nominal model parameters setting adopted for producing Fig. 4a.

Past experiments on a specific cell type (e.g. MSC) at various individual levels of oxygen below 20%, though all referring to as hypoxic conditions, have shown both higher and lower proliferation rates compared to that with normoxia (20%), hence leading to some ambiguity as to the role of hypoxia in cell proliferation. A compiled list of hypoxic literature experimental data for MSCs, converted in this work to the ratio of hypoxic proliferation rate to the rate at normoxia (20% oxygen), is shown in Fig. 4b [21–25, 56–64], as well as the hypoxia-to-normoxia ratio of the inverse of cell cycle commitment time (as an indicator of proliferation rate) obtained in simulation as presented in Fig. 4a. The rate ratios are shown on a logarithmic scale. The majority of the available data was collected under the range of in vitro intermediate or severe hypoxia, which limited the applicable range of comparison. Fig. 4b demonstrates that the simulation yielded results at the same level of magnitude as most of the literature findings. It also shows the lack of consensus between the experimental studies with respect to hypoxia-mediated effects on proliferation, possibly caused by the difference in cell line, culture condition and duration between these existing experimental studies. Future experimental work to produce a consistent data set that covers a sufficient range of oxygen level is thus needed to allow further validation and calibration of the model.

### Sensitivity of model parameters

**Fig. 5:**
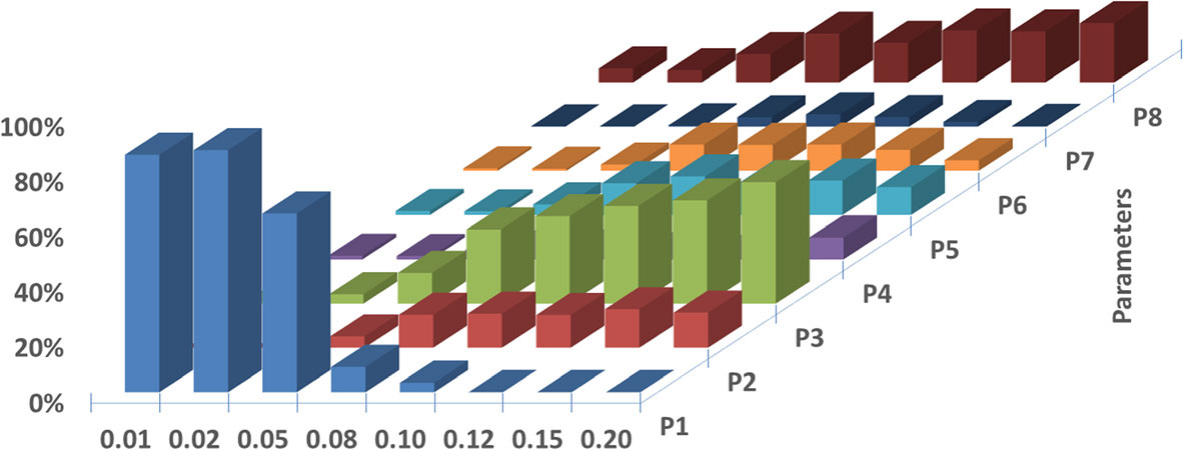
Derivative-based global sensitivity measure (DGSM) results for the selected model parameters. Relative parameter global sensitivity is shown as the calculated G-score on the z-axis. Eight different parameters have been evaluated under seven oxygen tensions (0.01, 0.02, 0.05, 0.08, 0.1, 0.15 and 0.2)

Global sensitivity analysis was conducted to test the robustness of the constructed model. The selected eight sensitive parameters are shown in Table 1. The two parameters for modelling ROS-mediated protein deactivation were investigated separately due to their special status. The converged G-scores across selected oxygen tensions are shown in Fig. 5. The effect of each parameter to cell cycle commitment time is sensitive to the applied oxygen level. Parameters P1, P3, P8 are identified as the top three parameters of significance. P1 dominates the proliferation response at oxygen tension less than 0.05, consistent with its role in positively regulating Hif1α level. Under mild hypoxia, Hif1α protein becomes negligible alongside with the diminishing importance of P1. P3 and P8 regulate Myc-facilitated E2F protein transcription and enhance Hif2α-Myc activity, respectively. Under mild hypoxia and normoxia, Hif1α-mediated Myc inactivation is liberated, allowing more Myc and Hif2α-Myc to take part in E2F transcription. This effect is reflected by the rising G-scores for P3 and P8 with an increase in oxygen tension. The results of DGSM highlight a few parameters that have a greater impact on simulation output, depending on the hypoxic oxygen level. The parameters with high sensitivities shall be prioritized in future model calibration and experimental validation.

**Table 1:** Parameters evaluated in DGSM study

P1	P2	P3	P4	P5	P6	P7	P8
b1	k’13	m13	k8	g_rb	m_e2f_rbp	d_cyce_pp	ε

### Variability in minimum CT and optimal oxygen level

**Fig. 6:**
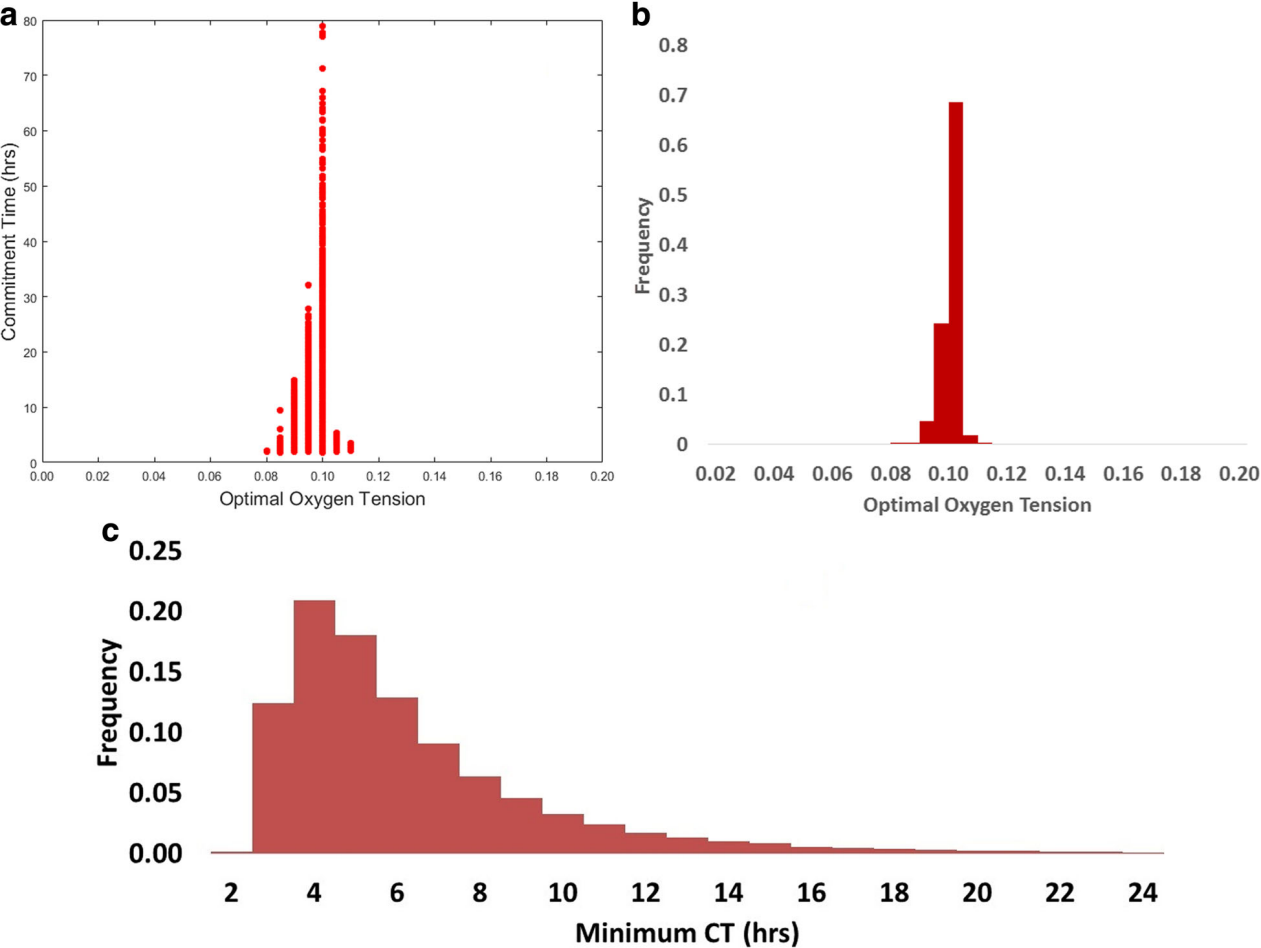
Results of QMC simulation with 39,000 sets of parameter values. **a.** Commitment time at optimal oxygen levels. Each point shows the minimum CT in each simulation. **b**. Frequency plot of optimal oxygen tension. **c**. Frequency plot of minimum commitment time (CT) across hypoxic oxygen tensions

Fig. 6a) and 6b) plot results of *O*_*2-optimal*_ and the corresponding minimum CT from the QMC simulation.

Fig. 6a) shows the large spread of CT at different optimal oxygen levels and Fig. 6b) confirms the existence of a concentrated range of the optimal oxygen level, which leads to the shortest commitment time. Despite the variation in parameter values, 98% of the simulated sample sets reported an optimal oxygen level within 9–10.5%, suggesting that the optimal oxygen level is relatively independent of the 8 highlighted parameters (P1-P8). This range is expected to change with different types of cells (cf. the later section on ROS-mediated protein deactivation), subject to their inherent and niche properties, but the convexity of the “U-shape” characterisation curve (as shown in Fig. 4) may be consistently preserved under the assumed mechanisms of HIFs and ROS, as shown by the QMC simulation results.

Fig. 6c) plots the frequency of minimum CT from each QMC run. The minimum CT appears to be sensitive to parameter settings, contrary to the narrow range observed for *O*_*2-optimal*_. A change in parameter values represents a quantitative shift in cellular behaviour, which directly affects the accumulation rates of cell cycle regulating proteins, hence the minimum CT. Nevertheless, approximately 90% of the minimal CT has been identified within the range of 3–11 h.

### Consistency of the shape of the characterisation curve

**Fig. 7:**
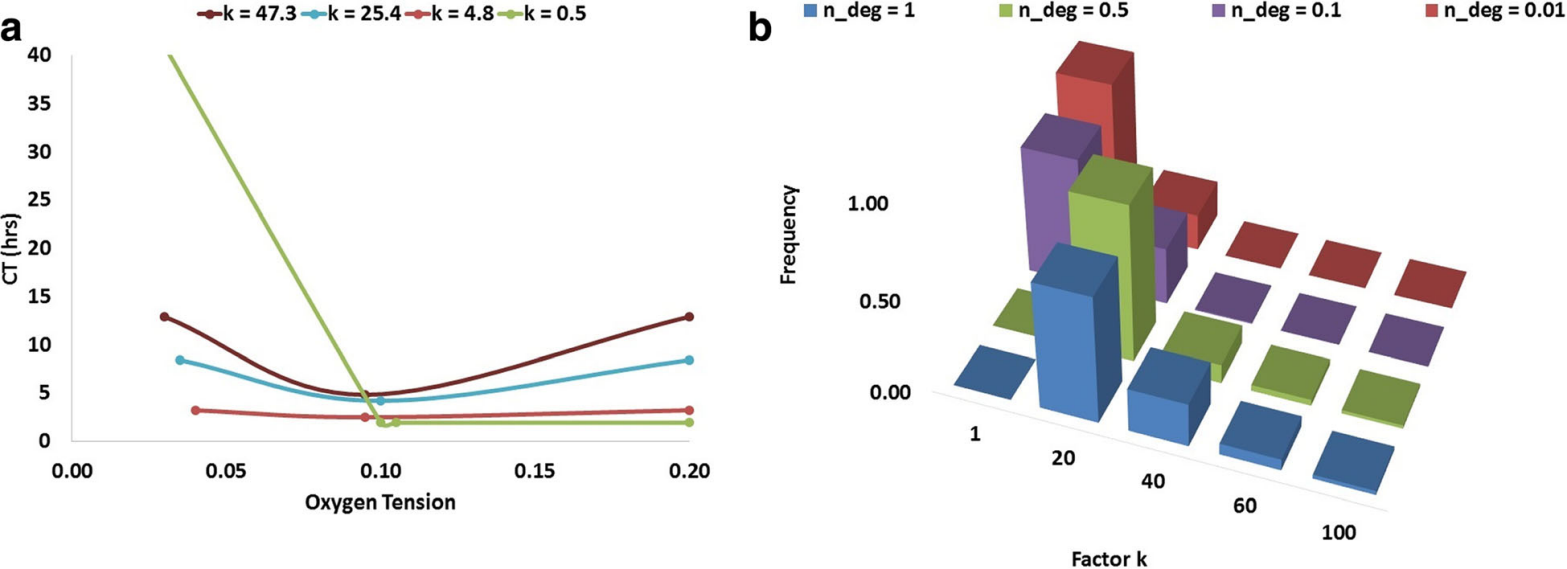
The shape of characterisation curves. **a** Sample curves for characterisation curves convexity factor k. The curves are selectively extracted from QMC simulation results for representation. **b** The frequency plot of the effect of parameter n_deg on factor k from compiled 39,000 sets of QMC simulation results

The QMC simulation results show that the convexity of characterisation (“CT – O_2_ level”) curve is preserved across the parameter sets. The shape of the characterisation curve was assessed with several exemplary cases, shown in Fig. 7a), via their respective k factors. A smaller k signifies a flatter parabola and a greater k indicates a higher oxygen dependency. For reference, the k value for the curve shown in Fig. 4a is 27.9. The frequency of k values for 39,000 QMC simulations (with *n_deg* = 1) is shown in Fig. 7b) (among results corresponding to other *n_deg* values, which are discussed in the later section). When *n_deg* (i.e. the parameter that controls protein deactivation) assumes this nominal value, the k values for over 99% of the simulated runs are within 1–40, representing the expected parabolic characterisation curves. The local derivatives of CT at *O*_*2-optimal*_ are relatively small, showing the robustness of cell cycle progression to a small perturbation in oxygen level in the vicinity of *O*_*2-optimal*_. This suggests that a rather tolerating range of oxygen control for cell growth may be accepted in designing the bioreactor control strategy.

If the value of k drops below 1, CT decreases significantly under severe and intermediate hypoxia with alleviated hypoxic stress, and then plateaus with negligible further reduction. The “U-shape” convexity is no longer present. This behaviour is only applicable in special scenarios, which are discussed in later sections.

### Impact of ROS-mediated general protein deactivation

Both *n_deg* and *[O*_*2*_*]*_*TH*_ are parameters associated with ROS-mediated general protein deactivation (shown in Eq. 16). Due to limited information and the distinct nature of these two parameters (they were fixed and not included in the global sensitivity analysis), separate sensitivity analysis was performed, to explore the impact of the extent of protein deactivation and the activation level.

**Fig. 8:**
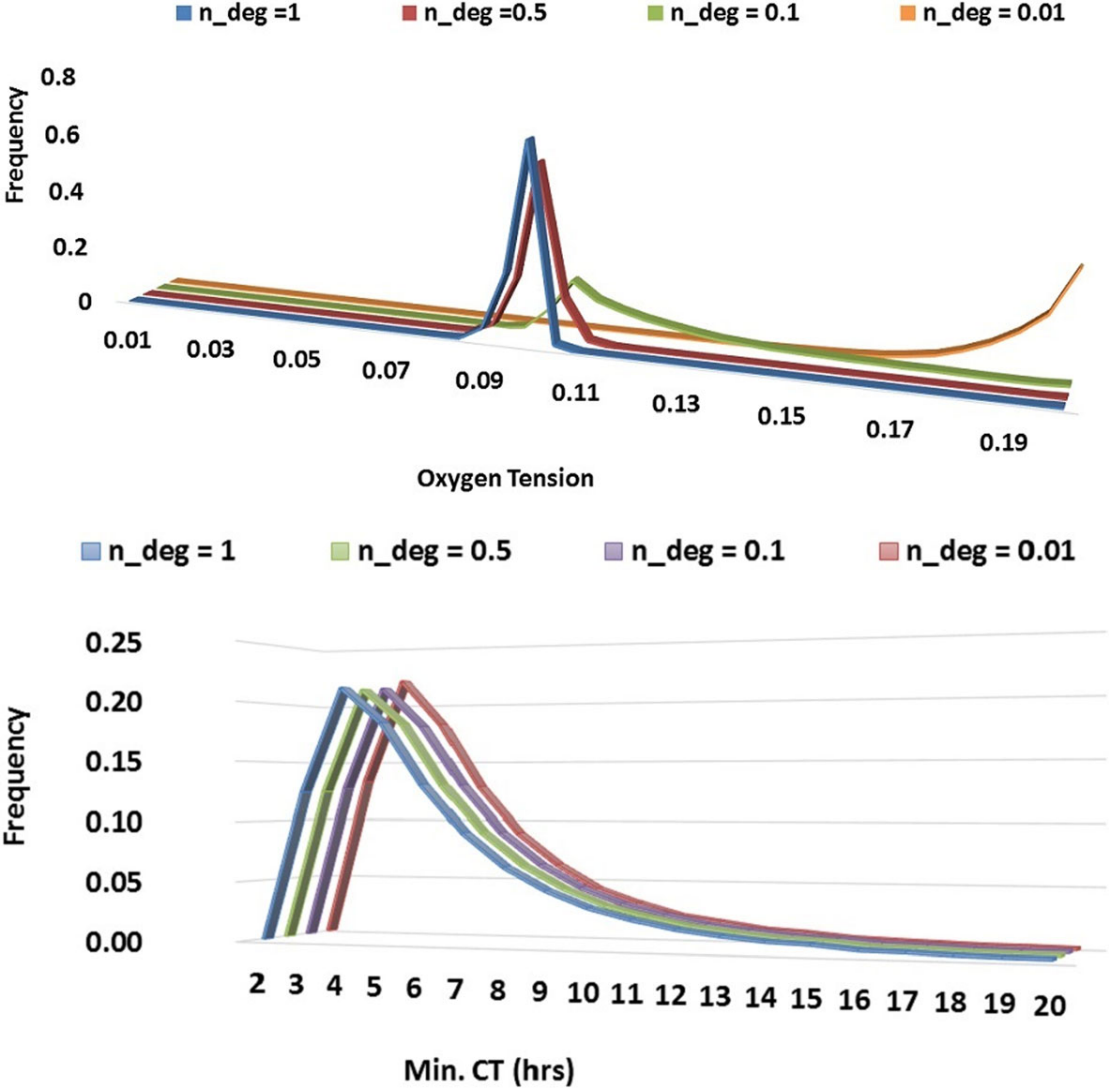
Impact of n_deg inferred from 39,000 sets of QMC simulation results. **a** The effect of n_deg on the frequency of optimal oxygen levels. **b** The effect of n_deg on the frequency of minimum CT

Values of *n_deg* between 0.01 and 1 have been studied. When *n_deg* = 0.01, ROS-mediated protein deactivation is negligible. Fig. 8a and b show that a smaller *n_deg* shifts the peaked optimal oxygen level towards normoxia with negligible impact on minimal CT. The reduction of *n_deg* also significantly alters the shape of the characterisation curve, as shown by an increase in the frequency of k values below one (Fig. 7b)). With *n_deg* below 0.1, the prolonged commitment to cell cycle under normoxia is mitigated, making higher oxygen tension more favourable for proliferation (Fig. 8a)).

**Fig. 9:**
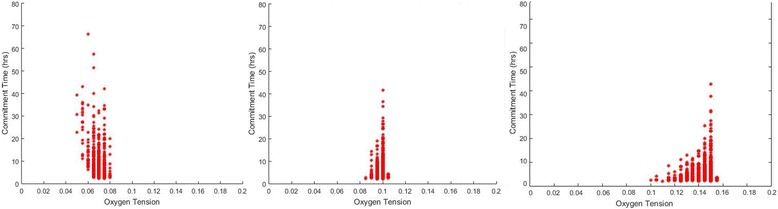
Simulations of various ROS-mediated protein deactivation activation oxygen level. The simulations were completed with activation oxygen level of 5% (left), 10% (central) and 15% (right). Each simulation was completed with the same 1000 QMC parameter value sets. The red dots represent the optimal oxygen level for each parameter set, showing the minimum commitment time in each simulation

The ROS activation level (*[O*_*2*_*]*_*TH*_) was varied from 5% to 15% reflecting oxygen levels potentially leading to different degrees of superoxide production that surpassed the inherent cellular degradation rate. Figure 9 plots the distribution of optimal oxygen level from QMC runs. O_2 TH_ is shown to have a significant impact on the oxygen level for quickest commitment to cell cycle; the level of dependency varies with the position of *[O*_*2*_*]*_*TH*_ within the range of hypoxic stress. Under intermediate hypoxia (*[O*_*2*_*]*_*TH*_ = 5%), other factors such as Hif1α and Hif2α-mediated responses share the control together with protein deactivation over cell cycle commitment time. Moving towards normoxia (*[O*_*2*_*]*_*TH*_ = 15%), proliferation responses from the two hypoxic factors are negligible. This further implies the significance of ROS-mediated protein deactivation on regulating commitment to cell cycle under the higher oxygen end of the hypoxic condition.

### Roles of parallel mechanisms on cell cycle commitment

A primary hypothesis during model building was the counteracting effects of Hif1α and Hif2α on commitment to cell cycle. From the simulation results, Hif1α competes with Hif2α for Myc binding and inactivates Myc for E2F production. Hif2α contributes consistently to E2F production individually and through paired Hif2α-Myc dimer under different gradients of simulated hypoxia. Under severe hypoxia, the pro-proliferation effect of Hif2α is to be overshadowed by that of Hif1α, which dominates the proliferation response and delays the progression towards meeting the cell cycle commitment requirement. As severe hypoxic stress is alleviated, the effect of Hif2α becomes more prominent and shortens CT.

**Fig. 10:**
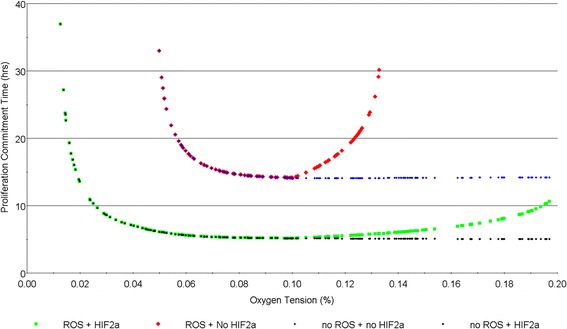
Comparative simulation results on the effect of ROS-mediated protein deactivation and Hif2α. The green dots indicate the nominal model parameter setting condition with ROS-mediated protein deactivation and the effects of Hif2α. The black dots account for Hif2α but not ROS-mediated protein deactivation. The blue and red simulations do not account for Hif2α. Simulations in red account for ROS-mediated deactivation, whereas simulation in blue does not. Note that in all cases Hif1α was assumed to function normally. The results were simulated with nominal parameter settings unless stated otherwise

Under oxygen levels greater than 10%, ROS-mediated general protein deactivation, when *[O*_*2*_*]*_*TH*_ is set to 10%, acts on all cell cycle regulators (Myc, Hif2α-Myc, E2F, etc.) and postpones the net accumulation of E2F. Hif2α-mediated E2F generation partially offsets the deactivation loss, when the effect of ROS is accounted for (with *n_deg* = 1). At *n_deg* = 0.01, ROS-mediated protein deactivation is negligible, Hif2α continues to facilitate commitment to cell cycle in the absence of any counteracting force under mild hypoxia and normoxia. A decrease in Hif2α concentration is expected with rising oxygen level beyond 5%, but the associated effect appeared to be mitigated by other dynamics embedded in the system. The peak oxygen level of Hif2α was found to have negligible effects (results not shown). Consequently, the flat-bottomed “L-shape” is observed when ROS-mediated protein deactivation is absent, making normoxia the favoured proliferation condition (Fig. 10).

Additionally, Hif2α is found to have dual effects on regulating E2F production and commitment to cell cycle. On the one hand, Hif2α-Myc dimer enhances E2F transcription activity. On the other hand, the displaced Myc is prevented from direct E2F transcription and cyclin D generation to free RB-bound E2F. Even though the two effects work in competition, both are needed to facilitate cell cycle commitment. This combined effect from the dual actions seems to be responsible for the decoupling of the optimal oxygen level (9–10.5%) with the peak Hif2α oxygen level (5%).

If ROS-mediated protein deactivation is absent, the optimal oxygen level extends to normoxia rather than falls within mild hypoxia. This is because the negative impact of Hif2α in Myc displacement plays a more prominent role when ROS-mediated protein deactivation is not in place, which leads to the “L-shape” characterisation curve. A characterisation curve with protein deactivation but without the presence of Hif2α can also be found in Fig. 10. In this case, the applicable range that leads to cell cycle commitment is much narrower, the minimal CT is greater, and the optimal oxygen level deviates from normoxia, as expected.

In summary, the simulation results indicate that Hif2α counteracts proliferation delay caused by ROS-mediated protein deactivation and Hif1α under normoxia and severe hypoxia, respectively, through facilitating E2F build-up. ROS-mediated protein deactivation may be a primary driver in causing proliferation delay under normoxia, which results in the observed hypoxia-mediated enhanced proliferation with certain cell lines.

## Discussion

Culturing MSCs under hypoxia in vitro has several observed advantages, such as stemness preservation and reduced senescence. This work has focused on the impact of hypoxic oxygen tension on the rate of cellular proliferation. Due to the resource-intensive process in experimentally testing all hypoxic oxygen tensions, most of the experimental work compare data from 1 to 3 hypoxic levels against the result acquired under normoxia (Fig. 4b); consistent experimental data on culturing MSCs over a full range of oxygen concentration have not yet been reported. This study incorporated a number of known mechanisms and hypotheses into a mathematical model to predict quantitatively the hypoxic response of cell cycle under conditions ranging from severe hypoxia to normoxia. In the following, we discuss about our model and the simulation results against data, knowledge and hypotheses given in the literature, highlight key implications, and also identify important limitations of this work to be addressed in the future.

Different reports have shown the counteracting effects between the Hifα isoforms on proliferation [4–6, 65, 66] and their functions in regulating many hypoxic functions [18, 67, 68]. To our knowledge, this study is the first attempt to integrate hypoxia sensing and cell cycle progression to capture the oxygen-dependent differential proliferation responses. The Myc protein was used to quantitatively couple and directly regulate key cell cycle regulating proteins. The simplified mechanism (Fig. 1) neglects the potential interactions from other Hifα-dependent [36, 69, 70] or Hifα-independent [26, 58, 71] hypoxic proliferation pathways. These pathways have been considered during model construction, but not included in the model. Some of their downstream regulators have been captured by the considered mechanisms, while others are either less significant or lack consensus in the literature. As confirmed knowledge about these and new pathways emerges, the first working model established in this study needs to be extended.

The simulation results, from applying a unified model over a wide range of oxygen levels, show that the effect of hypoxia on proliferation is dependent on its severity, due to the combined regulation originated by the two HIF factors and ROS-mediated protein deactivation. As such, the model has the potential to offer a reconciliation of the previously reported experimental results [1, 8, 10, 36, 69, 70, 72, 73]. Generally, a “U-shape” characterisation curve appears to be followed by the response of the commitment time to a change in in vitro hypoxic oxygen tension (Fig. 4a). The characterisation curve is subject to shift and contract, depending on the model parameter values, which in principle are dictated by properties of different cell lines and other culture conditions (medium, surface characteristics, etc.). However, under the assumptions of ROS-mediated protein deactivation, a specific, relatively narrow range of optimal oxygen tensions (9% - 10.5%) that would allow the quickest cell cycle commitment was consistently observed, despite the variation in parameter values (Fig. 6). This shows that, although the model has not been calibrated with a complete data set of any specific cell line hence is not suitable to make accurate predictions, a number of cell lines and culture systems may find their optimal oxygen concentration level within this range.

Global sensitivity analysis revealed the relative significance of selected parameters on impacting simulation output (Fig. 5). The parameter-specific dependency on oxygen tension was observed, which in some cases, were due to the nature of certain model equations (e.g., P1); other high impact parameters (e.g. P3 under in vitro normoxia) were a result of the interactions between multiple mechanisms modelled. The obtained ranking of parameters significance under different oxygen tensions shall be used to prioritize future work in experimental validation and parameter calibrations. Meanwhile, when comparing literature experimental findings, the sensitive parameters under the relevant oxygen level shall be accounted for to ensure a reflective comparison.

The net oxidative stress activation level (ROS-mediated protein deactivation threshold) has shown a significant impact over determining the range of the optimal oxygen levels (Fig. 9).In the complete absence of the ROS-mediated generic protein deactivation, the resultant “L-shape” characterisation curve is not an accurate reflection of the observed effect of hypoxia-enhanced proliferation [5, 8, 10, 11, 69, 72]. This behaviour, however, may be applicable for other types of cells with higher niche oxygen level, a projection to be confirmed by future experimental observations. Inherent anti-oxidant enzymes are expressed in stem cells to combat oxidative stress, however, with limited capacity, overwhelming ROS would lead to higher senescence and apoptosis [74, 75]. Bertolo et al. showed higher intracellular ROS led to longer division time in MSCs, which more than doubled in late passaged cells [76]. When exposed to the atmospheric oxygen level, which is much higher than MSCs niche condition, the levels of intracellular ROS and other reactive species could rise [48, 77–79]. The anti-oxidant capacity is believed to be cell type specific, which would result in a variation in the threshold and magnitude of ROS-mediated protein deactivation and consequently (as shown in our simulation) a shift in the range of optimal oxygen tension. This theoretical prediction however still needs to be validated through carefully designed experiments. Beyond what has been implemented in the current model, an increase in mitochondrial intracellular ROS production under severe hypoxia have also been reported [45, 80, 81]. The ROS also facilitates the stabilization of Hifα isoforms [51, 52, 82]. The impacts of those effects shall be considered in future studies.

To our knowledge, this work is the first one that mathematically models the role of Hif2α in cell cycle regulation. As a key hypoxia-regulating factor, it links to multiple functions in stem cell maintenance, pluripotency, and proliferation [5, 20, 36, 83–87]. In particular, various studies have reported the opposing effects of Hif2α with Hif1α [4, 6, 65, 66]. The simulation in this study showed that under intermediate hypoxia, Hif2α combats the negative effect of Hif1α on cell cycle progression; under normoxia, Hif2α can partially mitigate the delayed proliferation response caused by ROS-mediated protein deactivation. In this work, the Hif2α expression rate as a function of oxygen concentration was estimated by synthesizing various literature data; an improved measurement of intracellular Hif2α protein in MSCs through dedicated experimentation would yield a more accurate function. The dynamic variation of individual cell cycle regulating proteins during different stages of G1-phase progression provides insights into establishing a dynamic control strategy (i.e. for bioreactor operations) that may reduce the proliferation period via other controls, e.g. specific growth factors.

Several assumptions and simplifications were made to allow the construction of the current model, which require caution in interpreting the simulation results and, in many cases, point to important future work:

- The model focuses on the overall oxygen-dependent behaviour of cell cycle commitment and is not expected to be taken for making predictions at rather extreme conditions. In particular, under severe hypoxia pro-survival response in stem cells is expected to dominate and could result in quiescence to reduce DNA damage [9, 21, 88] despite a few reports showed the successful proliferation of MSCs under anoxic conditions [89, 90], which may be due to different factors as addressed in the original papers.
- The current model only estimates the time required for commitment to cell cycle in late-G1 phase. Future work will expand to include the entire cell cycle process to capture the rate of proliferation. The process of cell growth and metabolic uptake may also be considered in future work.
- More detailed Hif1α mechanistic models are available in the literature [28, 91] and may be incorporated to improve model accuracy and help identify cytokines to improve Hifα stabilization during in vitro culture.
- A key limitation of this study has been on data availability. The on-going growth in proteome and transcriptome studies poses the possibility of offering data with enhanced quantity and quality [92, 93]. With these newly available data, estimation of cell proliferation rate and identification of the optimal oxygen concentration and cell cycle commitment time could then be become more accurate.
- Finally, this single-cell model is intended to be integrated with a dynamic population model to capture the status of the overall cell pool in a bioreactor, addressing the growth kinetics and the heterogeneity of the MSCs pool.

## Conclusions

A single-cell mathematical model has been established to predict the time required for cell cycle commitment in a hypoxic environment as a proxy to proliferation rate. Incorporating known roles of HIF factors and introducing a hypothesis on ROS-mediated protein deactivation within a cohesive quantitative framework, the constructed model represents the first attempt to mechanistically capture these key factors in hypoxia sensing and cell cycle regulation. In particular, it implements a novel approach in using Myc to link oxygen sensing and G1 phase progression, allowing the differential responses of Hif1α and Hif2α to Myc activities and their respective oxygen-dependent availabilities to be quantified so as to predict cell proliferation status under different oxygen tensions.

Through extensive simulation that explored the parameters space using the quasi-Monte Carlo approach, the model revealed the preservation of optimal oxygen tension and the convexity of characterisation curve under the assumption of ROS-mediated protein deactivation, which suggests that the behaviours of severe hypoxia-mediated cell cycle arrest and mild hypoxia-mediated facilitated proliferation may be a rather common cellular response. Furthermore, the interactive effects of Hif2α on cell cycle progression with Hif1α and ROS-mediated protein deactivation appeared to act as opposing forces in regulating hypoxia-mediated proliferation.

The model developed in this work has been based on a synthesis of known biology and limited literature data. It offers a first vehicle for prediction cell cycle commitment overall a wide range of oxygen levels, which has not been covered by any existing experimental data set. While the order of magnitude of its predictions appears to be consistent with published data, it remains an important task to validate and calibrate the model with experimentation on specific cell lines, so that the model can eventually be used to suggest optimal operational strategies of bioreactors. The outstanding importance of certain model parameters revealed by the global sensitivity analysis, together with the high sensitivity of some mechanisms identified through the simulation studies, could offer useful guidance to the planning of future experimental work.

## Additional files

Additional file 1: Parameter values and initial conditions. (DOCX 23 kb)

Additional file 2: The effect of initial conditions on steady state E2F concentration. (DOCX 42 kb)

Additional file 3: Compiled hypoxic MSCs growth data. (DOCX 23 kb)

## Abbreviations

[O_2_]_TH_: Threshold oxygen level for ROS activation
CDKI: CDK inhibitors
CT: Cell cycle commitment time
DEG: ROS-mediated degradation mechanism at greater than 10% oxygen
DGSM: Derivative-based global sensitivity meansures
hESCs: Human Embryonic stem cells
HIF: Hypoxia inducible factor
MSCs: Mesenchymal stromal cells
O_2-optimal_: Optimal oxygen level
ODE: Ordinary differential equation
p/p: p21/p27, CDKI
QMC: Quasi-Monte carlo simulations
ROS: Reactive oxygen species
RP: Restriction point
