# Supplementary material for: Mathematical modelling of interacting mechanisms for hypoxia mediated cell cycle commitment for mesenchymal stromal cells
Source: BMC Syst Biol. 2018 Apr 2;12:35. doi: 10.1186/s12918-018-0560-3 (PMC5879778; doi:10.1186/s12918-018-0560-3)
Supplement: Supplementary file 1 — Parameter values and initial conditions. (DOCX 23 kb) [file 12918_2018_560_MOESM1_ESM.docx]

Mathematical modelling of interacting mechanisms for hypoxia mediated cell cycle commitment for Mesenchymal stromal cells

Supporting Information - Parameter values and initial conditions

Bo Zhang^1, 2^, Hua Ye^2^, Aidong Yang^1*^

^1^Department of Engineering Science, University of Oxford, Oxford, United Kingdom
^2^Institute of Biomedical Engineering, Department of Engineering Science, University of Oxford, Oxford, United Kingdom

S1: Tabulated nominal model parameter values and descriptions

| Parameter | Description | Reference/Note | Model Values |
| --- | --- | --- | --- |
| M1 | Hif1α multiplication constant | (1) | 0.000414 |
| b1 | Hif1α exponential constant |  | 9.395 |
| T1 | Hif1α normalizing oxygen level | (2) | 10% |
| M2 | Hif2α multiplication constant | (3, 4) | 0.20497 |
| b2 | Hif2α exponential constant |  | 0.44863 |
| kr3A | Hif1α-Myc dissociation | Estimated based on cyclin D – p21/p27 data (5) | 0.15 /hr |
| kf3A | Hif1α and Myc binding |  | 15 /uM/hr |
| kr3B | Hif2α- Myc dissociation |  | 0.15 /hr |
| kf3B | Hif2α and Myc binding |  | 15 /uM/hr |
| T2 | Hif2 normalizing oxygen level | (4) | 20% |
| m13 | E2F auto-regulation with Myc | (6) | 0.4 μM/h |
| k13 | E2F auto-regulation with Myc |  | 0.15 μM |
| k'13 | E2F auto-regulation with Myc |  | 0.15 μM |
| m_E2F | Myc induced E2F production |  | 0.003 μM/h |
| k_E2F | Myc induced E2F production |  | 0.15 μM |
| d_E2F | basal E2F degradation |  | 0.25 /hr |
| kf12 | cycE and p21/p27 association | (5, 7) | 18 /uM/h |
| kr6B | cycD-p/p dissociation | (5)same as in Rb-E2F binding | 0.18 /h |
| kr12 | cycE-p/p dissociation |  | 0.18 /h |
| g_cycD | basal cycD production | (5) 10% w serum | 0.045 μM/hr |
| k_3A | Hif1α direct inhibition | (5) assumed half of MYC-dependent cycD transcription strength | 0.07 μM |
| g_Rb | basal Rb synthesis | (5) | 0.06 μM/h |
| g_p/p | basal p21/p27 synthesis | (5) same as g_RB | 0.06 μM/h |
| d_p/p | basal p21/p27 degradation |  | 0.06 /h |
| d_cycd_pp | basal degradation of cycD-p/p | (5) same as d_cycE | 1.5 /hr |
| d_cyce_pp | basal degradation of cycE-p/p |  | 1.5 /hr |
| d_RbP | basal degradation of RbP | (5) same as d_RB | 0.06 /hr |
| d_e2f_rb | basal degradation of E2F-Rb | (5) same as d_E2F | 0.25 /hr |
| m_E2F-RbP | cycD phosphorylation of E2F-Rb | (5) | 18 /h |
| k_E2F-RbP | cycD phosphorylation of E2F-Rb |  | 0.92 μM |
| k8 | Rb and E2F association |  | 18 /uM/h |
| kf6B | cycD and p21/p27 association |  | 18 /uM/h |
| d_cycE | basal cycE degradation |  | 1.5 /h |
| k9 | E2F dependent production |  | 0.15 μM |
| m9 | E2F dependent production |  | 0.35 μM/h |
| k5_cycD | Myc-dependent cyclin D transcription |  | 0.15 μM |
| m5_cycD | Myc-dependent cyclin D transcription |  | 0.03 μM/h |
| d_cycD | basal cycD degradation |  | 1.5 /h |
| d_Rb | basal Rb degradation |  | 0.06 /h |
| k_cycD_RB | cycD phosphorylation of Rb |  | 0.92 μM |
| m_cycD_Rb | cycD phosphorylation of Rb |  | 18 /h |
| k_RbP | RbP dephosphorylation |  | 0.01 μM |
| m_RbP | RbP dephosphorylation |  | 5 μM/h |
| k_cycE_RbP | cyclin E-mediated hyper-phosphorylation of Rb |  | 0.92 μM |
| m_cycE_RbP | cyclin E-mediated hyper-phosphorylation of Rb |  | 18 /h |
| k_RbnP | RbnP de-phosphorylation to Rb |  | 0.01 μM |
| m_RbnP | RbnP de-phosphorylation to Rb |  | 5 μM/h |
| g_myc | basal Myc synthesis |  | 1 μM/h |
| d_myc | basal Myc degradation |  | 0.7 /h |
| k4 | Myc inhibition of p/p transcription | Estimated | 1 /uM |
| ε | relative activity strength of Hif2α-Myc over Myc |  | 1.1 |
| d_hif1_mm | basal degradation of Hif1α-Myc |  | 0.1 /hr |
| d_hif2_mm | basal degradation of Hif2α-Myc |  | 0.1 /hr |
| n_deg | ROS-facilitated degradation constant |  | 1 |

Tabulated Model Initial Conditions (5)

| Species | Initial Concentration (µM) |
| --- | --- |
| Hif1α-Myc; Hif2α-Myc; E2F; cycD-p/p; cycE-p/p; cycD; cycE; RbP; Rb-nP; Myc | 0 |
| E2F-RB | 0.25 |
| RB | 0.4 |
| p/p | 0.2 |

References

1. Jiang B-H, Semenza GL, Bauer C, Marti HH. Hypoxia-inducible factor 1 levels vary exponentially over a physiologically relevant range of O2 tension. American Journal of Physiology-Cell Physiology. 1996;271(4):C1172-C80.

2. Bracken CP, Fedele AO, Linke S, Balrak W, Lisy K, Whitelaw ML, et al. Cell-specific regulation of hypoxia-inducible factor (HIF)-1α and HIF-2α stabilization and transactivation in a graded oxygen environment. Journal of Biological Chemistry. 2006;281(32):22575-85.

3. Närvä E, Pursiheimo J-P, Laiho A, Rahkonen N, Emani MR, Viitala M, et al. Continuous hypoxic culturing of human embryonic stem cells enhances SSEA-3 and MYC levels. PloS one. 2013;8(11):e78847.

4. Forristal CE, Wright KL, Hanley NA, Oreffo RO, Houghton FD. Hypoxia inducible factors regulate pluripotency and proliferation in human embryonic stem cells cultured at reduced oxygen tensions. Reproduction. 2010;139(1):85-97.

5. Dong P, Maddali MV, Srimani JK, Thélot F, Nevins JR, Mathey-Prevot B, et al. Division of labour between Myc and G1 cyclins in cell cycle commitment and pace control. Nature communications. 2014;5.

6. Yao G, Tan C, West M, Nevins JR, You L. Origin of bistability underlying mammalian cell cycle entry. Molecular systems biology. 2011;7(1):485.

7. Novak B, Tyson JJ. A model for restriction point control of the mammalian cell cycle. Journal of theoretical biology. 2004;230(4):563-79.
