# Supplementary material for: Mathematical modelling of interacting mechanisms for hypoxia mediated cell cycle commitment for mesenchymal stromal cells
Source: BMC Syst Biol. 2018 Apr 2;12:35. doi: 10.1186/s12918-018-0560-3 (PMC5879778; doi:10.1186/s12918-018-0560-3)
Supplement: Supplementary file 2 — The effect of initial conditions on steady state E2F concentration. (DOCX 42 kb) [file 12918_2018_560_MOESM2_ESM.docx]

Mathematical modelling of interacting mechanisms for hypoxia mediated cell cycle commitment for Mesenchymal stromal cells

Supporting Information – The effect of initial conditions on steady state E2F concentration

Bo Zhang^1, 2^, Hua Ye^2^, Aidong Yang^1*^

^1^Department of Engineering Science, University of Oxford, Oxford, United Kingdom
^2^Institute of Biomedical Engineering, Department of Engineering Science, University of Oxford, Oxford, United Kingdom

The impact of different initial conditions was evaluated by running the simulations with different parameter value sets and initial conditions. The three key parameters (b1, m13, and ε) were selected for the analysis. Parameter values and initial conditions were varied by ±50% sampled by the Sobol sequence. For each parameter and initial condition set, the steady state E2F concentration was evaluated and compared to that predicted with the default initial conditions. The analysis was competed with 100 parameter value sets evaluated against 100 generated initial conditions (i.e. 10,000 scenarios in total). Out of the 10,000 scenarios, 198 scenarios (referred to as “outstanding scenarios”) were found to yield a steady state E2F concentration different from that obtained with the default initial conditions, which is less than 2% of the total cases tested. These data points are plotted in Figure S1 below. These outstanding scenarios plotted on the y-axis resulted in non-zero steady state E2F concentrations, however, the default initial conditions yielded E2F concentration of 0. Contrastingly, the scenarios plotted on the x-axis yielded non-zero steady state E2F concentrations but returned 0 with a new set of initial conditions. From this analysis, these outstanding scenarios only appeared to be caused by a switch between the initially quiescent cells and the commitment to proliferation, and vice versa.

It should be also noted that, out of 100 sets of parameter values tested, only 10 of them showed to lead to multiple steady states, 4 of which accounted for 164 of the 198 such scenarios. In terms of oxygen levels, a large portion of the scenarios with multiple steady states was observed for the lower oxygen levels (severe hypoxia) and close to normoxia.


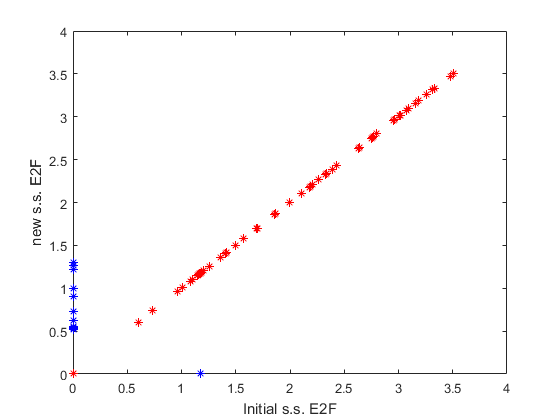


Figure S1: Simulation results on the steady state E2F concentrations from the simulation results for different parameter value sets and initial conditions. The 9802 simulated results that yielded the same steady state E2F concentrations are shown in red. The 198 cases that showed discrepancies on the steady state E2F levels are plotted in blue.
