# Supplementary material for: Mathematical modelling of interacting mechanisms for hypoxia mediated cell cycle commitment for mesenchymal stromal cells
Source: BMC Syst Biol. 2018 Apr 2;12:35. doi: 10.1186/s12918-018-0560-3 (PMC5879778; doi:10.1186/s12918-018-0560-3)
Supplement: Supplementary file 3 — Compiled hypoxic MSCs growth data. (DOCX 23 kb) [file 12918_2018_560_MOESM3_ESM.docx]

Mathematical modelling of interacting mechanisms for hypoxia mediated cell cycle commitment for Mesenchymal stromal cells

Supporting Information – Compiled hypoxic MSCs growth data

Bo Zhang^1, 2^, Hua Ye^2^, Aidong Yang^1*^

^1^Department of Engineering Science, University of Oxford, Oxford, United Kingdom
^2^Institute of Biomedical Engineering, Department of Engineering Science, University of Oxford, Oxford, United Kingdom

Table S2: The compiled hypoxic growth data from the literature for MSCs. The table below corresponds to Figure 4.

| Hypoxic level | Figure 4 reference | Reference |
| --- | --- | --- |
| 2% | S1 | [1] |
| 1% | S2 | [2] |
| 2% | S3 | [3] |
| 2% | S4 | [4] |
| 1% | S5 | [5] |
| 0.50% | S6 | [6] |
| 2.50% |  |  |
| 0.50% |  |  |
| 2.50% |  |  |
| 1% | S7 | [7] |
| 3% |  |  |
| 5% |  |  |
| 10% |  |  |
| 1% | S8 | [8] |
| 1% |  |  |
| 2% | S9 | [1] |
| 5% | S10 | [9] |
| 2% | S11 | [10] |
| 5% | S12 | [11] |
| 1% | S13 | [12] |
| 1% | S14 | [13] |
| 5% |  |  |
| 1% |  |  |
| 5% |  |  |
| 2% | S15 | [14] |

1. Grayson WL, Zhao F, Bunnell B, Ma T: **Hypoxia enhances proliferation and tissue formation of human mesenchymal stem cells.** *Biochemical and biophysical research communications* 2007, **358:**948-953.

2. Fotia C, Massa A, Boriani F, Baldini N, Granchi D: **Hypoxia enhances proliferation and stemness of human adipose-derived mesenchymal stem cells.** *Cytotechnology* 2015, **67:**1073-1084.

3. Grayson WL, Zhao F, Izadpanah R, Bunnell B, Ma T: **Effects of hypoxia on human mesenchymal stem cell expansion and plasticity in 3D constructs.** *Journal of cellular physiology* 2006, **207:**331-339.

4. Dos Santos F, Andrade PZ, Boura JS, Abecasis MM, Da Silva CL, Cabral J: **Ex vivo expansion of human mesenchymal stem cells: a more effective cell proliferation kinetics and metabolism under hypoxia.** *Journal of cellular physiology* 2010, **223:**27-35.

5. Hung SP, Ho JH, Shih YRV, Lo T, Lee OK: **Hypoxia promotes proliferation and osteogenic differentiation potentials of human mesenchymal stem cells.** *Journal of Orthopaedic Research* 2012, **30:**260-266.

6. Sharma S, Bhonde R: **Mesenchymal stromal cells are genetically stable under a hostile in vivo–like scenario as revealed by in vitro micronucleus test.** *Cytotherapy* 2015, **17:**1384-1395.

7. D'Ippolito G, Diabira S, Howard GA, Roos BA, Schiller PC: **Low oxygen tension inhibits osteogenic differentiation and enhances stemness of human MIAMI cells.** *Bone* 2006, **39:**513-522.

8. Das R, Jahr H, van Osch GJ, Farrell E: **The role of hypoxia in bone marrow–derived mesenchymal stem cells: considerations for regenerative medicine approaches.** *Tissue Engineering Part B: Reviews* 2009, **16:**159-168.

9. Kurosawa H, Kimura M, Noda T, Amano Y: **Effect of oxygen on in vitro differentiation of mouse embryonic stem cells.** *Journal of Bioscience and Bioengineering* 2006, **101:**26-30.

10. Sheng L, Mao X, Yu Q, Yu D: **Effect of the PI3K/AKT signaling pathway on hypoxia‑induced proliferation and differentiation of bone marrow‑derived mesenchymal stem cells.** *Experimental and Therapeutic Medicine* 2017, **13:**55-62.

11. Peng L, Shu X, Lang C, Yu X: **Effects of hypoxia on proliferation of human cord blood-derived mesenchymal stem cells.** *Cytotechnology* 2016, **68:**1615-1622.

12. Tsai C-C, Chen Y-J, Yew T-L, Chen L-L, Wang J-Y, Chiu C-H, Hung S-C: **Hypoxia inhibits senescence and maintains mesenchymal stem cell properties through down-regulation of E2A-p21 by HIF-TWIST.** *Blood* 2011, **117:**459-469.

13. Chung D-J, Hayashi K, Toupadakis CA, Wong A, Yellowley CE: **Osteogenic proliferation and differentiation of canine bone marrow and adipose tissue derived mesenchymal stromal cells and the influence of hypoxia.** *Research in veterinary science* 2012, **92:**66-75.

14. Valorani M, Montelatici E, Germani A, Biddle A, D'alessandro D, Strollo R, Patrizi M, Lazzari L, Nye E, Otto W: **Pre‐culturing human adipose tissue mesenchymal stem cells under hypoxia increases their adipogenic and osteogenic differentiation potentials.** *Cell Proliferation* 2012, **45:**225-238.
